# Sociodemographic Differences in the Dietary Quality of Food-at-Home Acquisitions and Purchases among Participants in the U.S. Nationally Representative Food Acquisition and Purchase Survey (FoodAPS)

**DOI:** 10.3390/nu12082354

**Published:** 2020-08-07

**Authors:** Maya K. Vadiveloo, Haley W. Parker, Filippa Juul, Niyati Parekh

**Affiliations:** 1 Department of Nutrition and Food Sciences, University of Rhode Island, Kingston, RI 02881, USA; haleyparker@uri.edu; 2 School of Global Public Health, New York University, New York, NY 10012, USA; fcj211@nyu.edu (F.J.); np31@nyu.edu (N.P.); 3 New York University School of Medicine, New York, NY 10016, USA

**Keywords:** grocery purchase quality, FoodAPS, Food Acquisition and Purchase Survey, food purchases, sociodemographic disparities, diet quality, Healthy Eating Index

## Abstract

Insufficient research has explored whether sociodemographic differences in self-reported, individual-level diet quality are similarly reflected by grocery purchase quality. This cross-sectional analysis of *n* = 3961 U.S. households from the nationally representative Food Acquisition and Purchase Survey (FoodAPS) compared Healthy Eating Index (HEI)-2015 scores from 1 week of food-at-home acquisitions across self-reported demographic factors (race/ethnicity, Supplemental Nutrition Assistance Program (SNAP) participation, food security, and household-level obesity status). Multivariable-adjusted, survey-weighted regression models compared household HEI-2015 scores across sociodemographic groups. Respondents were primarily White and female, with a mean age of 50.6 years, 14.0% were food insecure, and 12.7% were SNAP-participating. Mean HEI-2015 scores were 54.7; scores differed across all sociodemographic exposures (*p* < 0.05). Interactions (*p* < 0.1) were detected between SNAP participation and (1) food insecurity and (2) household-level obesity, and race/ethnicity and (1) household-level obesity. HEI-2015 scores were higher among food secure, non-SNAP households than among food insecure, SNAP-participating households (53.9 ± 0.5 vs. 50.3 ± 0.7, *p* = 0.007); non-SNAP households without obesity had significantly higher HEI-2015 scores than other households. Household-level obesity was associated with lower HEI-2015 scores in White (50.8 ± 0.5 vs. 52.5 ± 0.7, *p* = 0.046) and Black (48.8 ± 1.5 vs. 53.1 ± 1.4, *p* = 0.018) but not Hispanic households (54.4 ± 1.0 vs. 52.2 ± 1.2, *p* = 0.21). Sociodemographic disparities in household HEI-2015 scores were consistent with previous research on individual-level diet quality.

## 1. Introduction

Improving diet and reducing diet-related disparities consistently observed across socioeconomic, racial/ethnic, and body weight groups [1,2] is urgently needed to reduce the burden of diet-related diseases in the U.S. [3]. While there are numerous efforts to improve dietary intake, routine measurement of diet is challenging despite its importance in evaluating the effect of public health interventions [4,5]. Commonly used sources of dietary data rely on retrospective recalls of self-reported intake, which are impacted by systemic and random errors related to portion size estimation and omission [6,7]. Therefore, novel dietary data sources and data capture methods are needed.

Interestingly, grocery purchase data may expand opportunities for ongoing dietary surveillance and evaluation of interventions targeting diet-related disparities [8]. While grocery purchases—particularly at the household-level—do not perfectly reflect individual-level intake, grocery purchase sales data are strongly associated with self-reported, individual-level nutrient intake [9,10], and the dietary quality of foods purchased and the dietary quality of foods consumed are correlated [11]. Furthermore, as an alternative source of dietary data, grocery acquisition data may circumvent well-documented limitations of self-reported intake data while providing a lens into often unmeasured characteristics of local food environments (e.g., types of retailers, cost of living, access to transportation), which influence food availability in different communities [12,13,14,15,16,17,18].

Despite the potential utility of household grocery purchase data as an adjuvant diet assessment method, research has not yet established whether socioeconomic, racial/ethnic, and body weight-based diet quality disparities exist in household-level grocery purchase quality similarly to those consistently observed in individual-level self-reported intake [1,2]. In the U.S., poor diet quality disproportionately impacts racial/ethnic minorities, people with low socioeconomic status, and people with overweight and obesity [1,2]. One of the most consistent U.S. diet quality disparities is present among Black populations who on average have lower diet quality than their White counterparts [19,20,21,22]. Poor diet quality is also consistently observed in groups with low socioeconomic status (e.g., lower household income, food insecurity, and/or participation in the Supplemental Nutrition Assistance Program (SNAP)) and with overweight and obesity [1,23].

**Figure 1:**
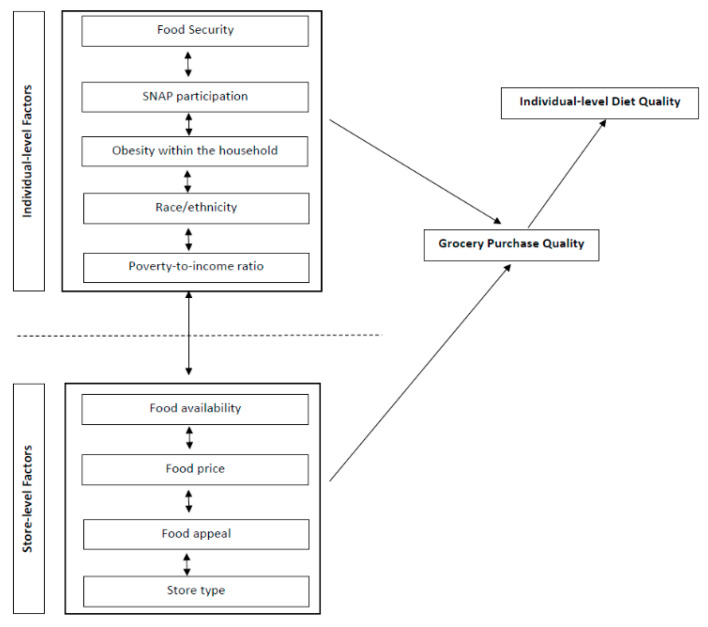
Conceptual framework depicts the proposed relationship between socioeconomic and demographic factors at the individual- and environmental-level that shape the dietary quality of a household’s grocery purchases and subsequent immediate home food environment and individual-level diet quality.

Since groups experiencing diet quality disparities frequently overlap (e.g., race/ethnicity is highly associated with socioeconomic status [19]), it is important to consider the synergistic influence of co-occurring disparities on food procurement and intake [24,25,26,27]. As depicted in Figure 1, grocery acquisitions and subsequent intake are influenced by interrelated sociodemographic factors as well as factors in the food environment. Describing the diet quality of household food-at-home (FAH) acquisitions among sociodemographic groups (i.e., race, income, SNAP participation, food insecurity, obesity) may inform whether sociodemographic dietary risk factors consistently observed in individual-level studies [1,2] are reflected in purchasing and acquisition data. Furthermore, because grocery procurement is influenced by access and availability in the broader food environment, examining disparities in grocery purchase quality may help contextualize individual-level sociodemographic disparities in dietary quality and diet-related outcomes.

Therefore, the objectives of this study are twofold. First, this study will describe racial/ethnic, socioeconomic, and weight-based differences in household FAH acquisition diet quality (measured using the Healthy Eating Index (HEI)-2015) utilizing a nationally representative National Household Food Acquisition and Purchase Survey (FoodAPS) [28]. Second, this study will descriptively evaluate whether these differences are consistent with sociodemographic disparities in diet quality observed at the individual level, providing insight into the utility of FAH acquisition diet assessment data [29,30]. The authors hypothesize that nationally representative FAH acquisition data will reveal diet quality disparities across different racial/ethnic, socioeconomic, and weight-based groups similar to those identified in individual-level dietary data.

## 2. Methods

### 2.1. Data Source

Publicly available food acquisition and purchasing data from the United States Department of Agriculture’s FoodAPS study, exempt from Institutional Review Board review, were used. The purpose of FoodAPS was to provide comprehensive data regarding U.S. households’ food acquisitions and purchases for research on determinants of food acquisition decisions and to inform policymaking regarding health, obesity, hunger, and nutrition assistance programs [31]. Data were collected between April 2012 and January 2013 from a nationally representative, cross-sectional sample of 4826 U.S. households, using a multistage sample design [31].

In recruited households, the main food shopper or meal planner was designated as the primary respondent (PR) who, on behalf of the household, answered in-person interview questions regarding sociodemographic characteristics, health, and food security [31]. During the interview, the PR was trained to use a hand scanner and food books to record all food acquisitions over a 7 day period [31]. Purchasing and acquisition events were classified by the PR into two exclusive categories; (1) Food at home (FAH): groceries, foods, and drinks that were brought home and used to prepare meals consumed at home or elsewhere (e.g., a packed lunch prepared at home and consumed at work) and (2) Food away from home (FAFH): meals, snacks, and drinks from outside the home, including items purchased and consumed outside of the home as well as prepared foods (e.g., take-out, delivery) consumed at home [31]. The present study focused exclusively on FAH items because current technology makes automated collection of grocery purchase data feasible; detecting similar patterns between FAH data and self-report would provide important insight into novel approaches for monitoring population diet quality. Quantity acquired was missing for 14.3% of FAH items; compared to convenience foods and grocery store purchases, quantities were more commonly missing for items such as meats and vegetables acquired at farmers markets or through own production (e.g., hunting, gardening). FoodAPS researchers imputed gram quantity data for HEI calculations based on information about food items, price, the stores from which they were acquired, and household characteristics [32]. The FoodAPS survey design and data collection process is described in detail in the FoodAPS User’s Guide [33].

### 2.2. Analytical Sample

**Figure 2:**
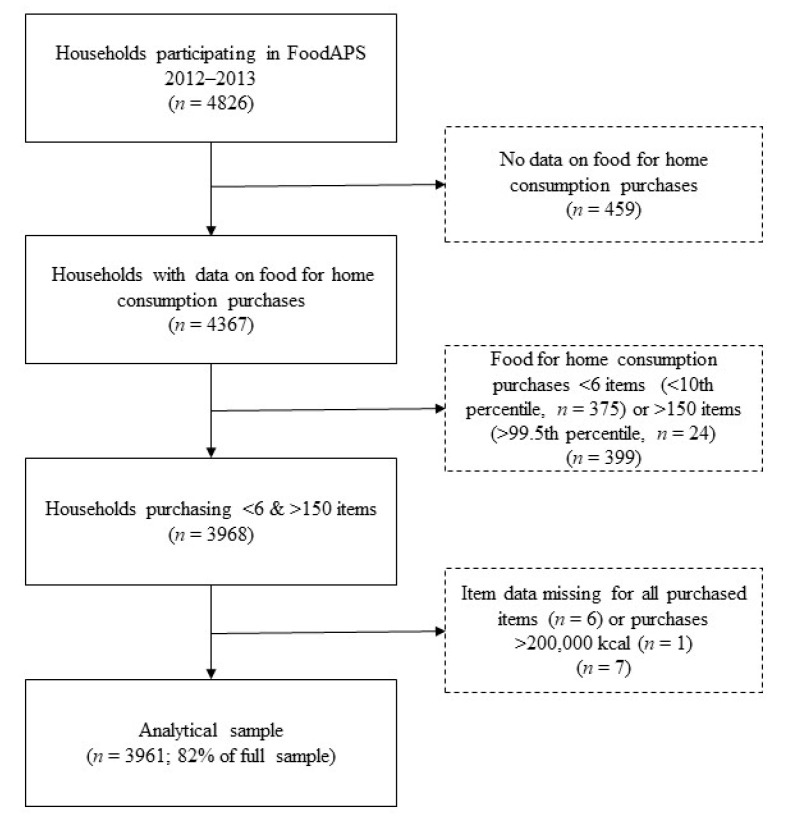
Flowchart of the creation of the analytical sample of households participating in the National Household Food Acquisition and Purchase Survey 2012–2013. Reproduced with permission from Juul et al., 2019 [32].

Of the 4826 households participating in FoodAPS, a total of 4367 households had data on FAH acquisitions and were eligible for inclusion in the current study. Based on the sample distribution and consistent with previous analyses [34], households reporting FAH acquisitions unlikely to be representative of usual weekly FAH acquisitions, defined as <6 items (<10th percentile, *n* = 375) or >150 items (>99.5th percentile, *n* = 24), were excluded. An additional six households were excluded that only acquired items that could not be identified, and one household was excluded as an outlier because total energy acquired was 2,716,827 kcal, i.e., >15 times greater compared to the household acquiring the second greatest amount of energy (177,648 kcal). The final analytical sample included 3961 households, visualized in Figure 2 [34].

### 2.3. Healthy Eating Index 2015

The HEI-2015 was used to assess diet quality of FAH acquisitions, reflecting household-level adherence to the 2015–2020 Dietary Guidelines for Americans [29,35]. The density-based scoring (i.e., food group or nutrient intake/1000 kcals) enables the HEI-2015 to be applied to standard intake data as well as to alternative food-related data sources (e.g., menus, sales fliers, food supplies), provided that food items can be linked to standard reference values via the Food and Nutrient Database for Dietary Studies [36,37]. The HEI-2015 has a maximum score of 100 and is the sum of nine adequacy and four moderation component scores, the latter being reverse coded (i.e., lower intake equates to a higher score) [38]. Higher scores indicate better diet quality. Similar to some previous research [30], the HEI-2015 was selected over the HEI-2010 [8,39] to evaluate concordance of FAH acquisitions with the most current dietary guidance. Research suggests that the HEI-2015 scoring is mostly concordant with the 2010–2015 Dietary Guidelines, but further emphasizes plant-based proteins and limiting added sugars [40].

The HEI-2015 component and total scores were calculated for FAH acquisitions for each household using the publicly available statistical code from the Division of Cancer Control and Population Sciences of the National Cancer Institute for the simple HEI algorithm scoring method [41]. Households were categorized into three HEI-2015 total score groups, <40 (low), 40–59 (medium), and ≥60 (high) [42], as the sample distribution (range = 11.7–98.3, median = 51.5, interquartile range = 43.1–60.9) did not allow for using other proposed HEI grades for evaluating diet quality [43].

### 2.4. Exposures

One objective of this study was to describe the overall diet quality of household FAH acquisitions across sociodemographic groups (i.e., race/ethnicity, SNAP participation, food insecurity, and obesity). Consistent with analyses examining sociodemographic predictors of individual-level diet quality, this study examined associations between HEI-2015 scores and the PR’s race/ethnicity (non-Hispanic White (NHW), non-Hispanic Black (NHB), Hispanic, and Other/mixed race/ethnicity), the household’s current SNAP participation (U.S. program designed to alleviate food insecurity for households ≤ 130% of the federal poverty level), food security status (very low/low/marginal/high food security from a modified U.S. Department of Agriculture 30 day adult food security scale [44] was dichotomized as food secure (marginal/high food security) and food insecure (very low/low food security)), household-level obesity (<1 vs. ≥1 household member(s), where body mass index (BMI) calculated from PR-reported height and weight was ≥30 [45]), and household poverty income ratio [46] (PIR; categorized as <130%, 130–349%, ≥350%) [47].

### 2.5. Covariates

A theory- and data-based model-building approach was used. Potential covariates were selected from the literature and evaluated for inclusion in multivariable-adjusted models. All covariates considered were self-reported by the PR and included PR-level variables for age, education, self-reported health status, and self-reported healthfulness of the household’s diet; as well as household-level variables for smoking, obesity [48,49,50], number of household members, urban vs. rural, number of FAH items, and calories acquired. Furthermore, primary exposure variables (i.e., race/ethnicity, SNAP participation, food security status, PIR (continuous covariate), and household-level obesity) were evaluated as possible covariates when not analyzed as the primary exposure variable. Excluding age and sex, which were retained regardless of statistical considerations, covariates were singly added to univariate models (and stratified models when effect modification was detected). Retention was based on the covariate’s significance in the model (*p* < 0.05) and impact on the variance explained (i.e., R^2^); if R^2^ increased by >150% when the covariate was added to the unadjusted model, it was retained.

### 2.6. Statistical Analysis

Descriptive statistics (means, standard deviations, and frequencies) were calculated for sociodemographic characteristics within the overall analytic sample and according to HEI-2015 group (<40, 40–59, and ≥60 pts) [42]. Statistical differences in HEI-2015 groups were examined using Pearson’s chi-square tests for categorical variables and unadjusted linear regression for continuous characteristics, treating the HEI-2015 group as an ordinal variable.

Survey-weighted linear regression was used to calculate unadjusted and multivariable-adjusted least square means and standard errors of the HEI-2015 total and component scores. Beta coefficients with 95% confidence intervals were also calculated. Effect modification in unadjusted models between each of the main exposures (e.g., SNAP participation, food security status, race/ethnicity, presence of obesity in household, and PIR) were evaluated using a significance threshold of <0.1. The coefficient of variation (ratio of the standard error (SE) to the mean) was calculated to assess the reliability of the survey-weighted score estimates. Estimates were considered reliable by the Economic Research Service (Washington, DC, USA, X. Zhang, e-mail communication, July 2018) if the coefficient of variation did not exceed 0.3.

All analyses were conducted in SAS 9.4 (SAS Institute, Inc., Cary, NC, USA) [51]. The FoodAPS sample weights and Taylor series linearization method for variance estimation were used in all analyses to account for the complex sample design of the survey. A *p*-value of 0.05 or lower was considered significant. Tukey adjustment and planned comparisons were used to correct for multiple post hoc comparisons between sociodemographic groups [52]. Pairwise post hoc comparisons were made in all analyses excluding analyses stratified by race/ethnicity, in the case of planned comparisons (within racial/ethnic groups and across racial/ethnic groups using NHW as the reference group).

## 3. Results

### 3.1. Descriptive Characteristics

The demographic and socioeconomic characteristics of the analytic sample according to HEI-2015 score group (<40, 40–59, and ≥60) are presented in Table 1. Household PRs were on average 50.6 years of age, predominantly NHW (70.3%), female (70.2%), and had obtained a high school degree or attended some college (57.8%). While most households (83.1%) had an income ≥ 130% of the federal poverty threshold, 14.0% were food insecure and 12.7% received SNAP benefits.

There was a significant linear trend between HEI-2015 score group and household size, number of children, and age of the PR; households with a HEI-2015 total score ≥ 60 were smaller (*p* = 0.005), had fewer children (*p* < 0.001) who were older (*p* = 0.015), were more likely NHW or Other race/ethnicity, and had higher educational attainment (*p* < 0.001) than households with a HEI-2015 score < 40. Conversely, households with a HEI-2015 total score < 40 were more likely to be <130% of the federal poverty threshold (*p* < 0.001), SNAP participating (*p* < 0.001), food insecure (*p* < 0.001), have household-level obesity (*p* < 0.001), smokers within the household (*p* < 0.001), and report fair or poor PR health status (*p* < 0.001) than households with a HEI-2015 score ≥ 60. Households with higher HEI-2015 total scores acquired a comparable total number of kilocalories but significantly more items (*p* < 0.001) compared to households with lower diet quality.

### 3.2. Unadjusted HEI-2015 Scores across Sociodemographic Groups

Unadjusted mean HEI-2015 total and component scores for all households and mean HEI-2015 total score across the fivehousehold demographic and socioeconomic exposure variables are presented in Table 2. The average HEI-2015 total score for FAH acquisitions was 54.7 ± 0.4 points. Overall, households were furthest from meeting recommendations for whole grains (2.8 out of 10 points), total vegetables (1.9 out of 5 points), and seafood and plant proteins (2.4 out of 5 points), and closest to meeting guidelines for total protein foods (3.6 out of 5 points), sodium (6.8 out of 10 points), and refined grains (6.7 out of 10 points).

All sociodemographic factors and primary exposure variables (race/ethnicity, food security status, SNAP participation, PIR, and household-level obesity) were significant predictors of HEI-2015 total scores. Compared to NHW households (54.9 ± 0.5 points), mean HEI-2015 total scores were lower among NHB households (51.5 ± 1.3 points, *p* < 0.008) and higher among households of Other race/ethnicity (58.1 ± 1.1 points, *p* = 0.02). HEI-2015 total scores did not differ between Hispanic (54.1 ± 0.7 points) and NHW households. FAH diet quality was lower among food insecure households compared to food secure households (50.1 ± 0.6 vs. 55.4 ± 0.4 points, *p* < 0.001), among SNAP participating households compared to non-SNAP households (49.1 ± 0.5 vs. 55.5 ± 0.4 points), and among those with obesity at the household-level compared to those without obesity (52.8 ± 0.6 vs. 56.2 ± 0.5 points, *p* < 0.001). Diet quality was higher among households with a PIR ≥ 350% (57.1 ± 0.6 points), compared to households with a PIR of 130–349% (53.8 ± 0.6 points) and <130% (50.7 ± 0.7 points, *p* < 0.001).

**Figure 3:**
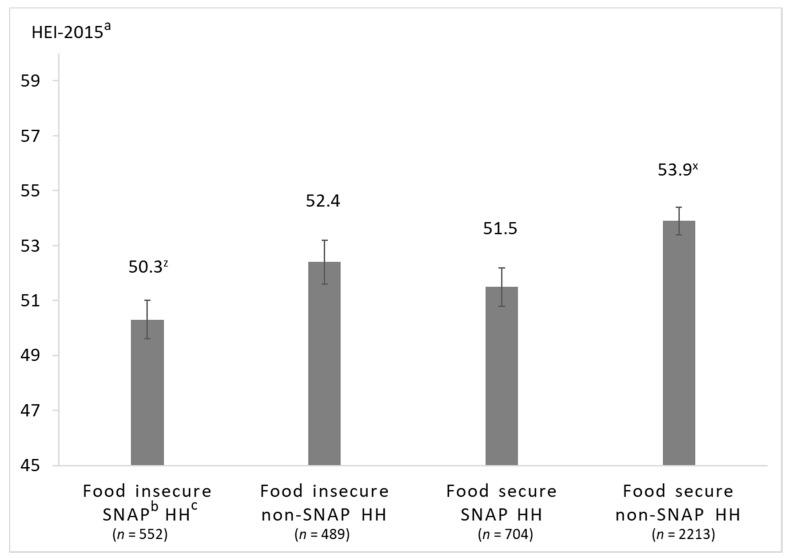
Multivariable-adjusted least square means of total HEI-2015 score of household grocery purchases among households participating in the National Household Food Acquisition and Purchase Survey 2012–2013, (*n* = 3958), stratified by food security status and SNAP-participation. ^a^ Healthy Eating Index 2015; ^b^ Supplemental Nutrition Assistance Program; ^c^ household. Adjusted for age of the primary respondent, family income-to-poverty ratio, any smoker in the household, and education level of the primary respondent. Between-group differences were determined by post hoc tests using Tukey’s method, testing all pairwise comparisons. Scores with different superscripted letters (e.g., x and z) indicate significant post hoc differences between groups (*p* < 0.05).

**Figure 4:**
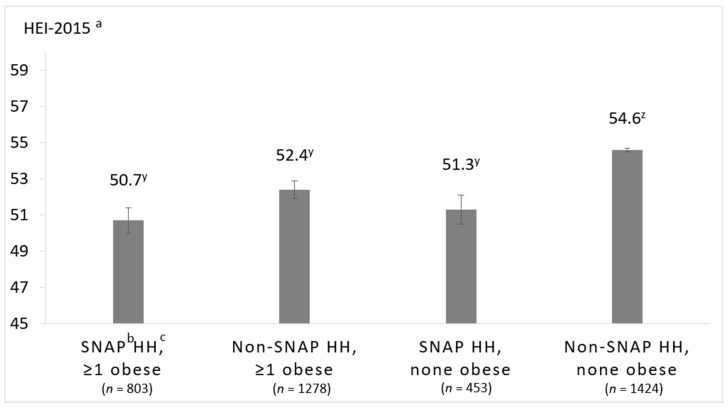
Multivariable-adjusted least square means of total HEI-2015 score of household grocery purchases of households participating in the National Household Food Acquisition and Purchase Survey 2012–2013, (*n* = 3958), stratified by weight status (none obese vs. ≥1 obese, with obesity defined as body mass index (BMI) ≥ 30 kg/m^2^) and SNAP-participation. ^a^ Healthy Eating Index 2015, ^b^ Supplemental Nutrition Assistance Program, ^c^ household. Adjusted for age of the primary respondent, family income-to-poverty ratio, any smoker in the household, and education level of the primary respondent. Between-group differences were determined by post hoc tests using Tukey’s method, testing all pairwise comparisons. Scores with different superscripted letters (e.g., y and z) indicate significant post hoc differences between groups (*p* < 0.05).

**Figure 5:**
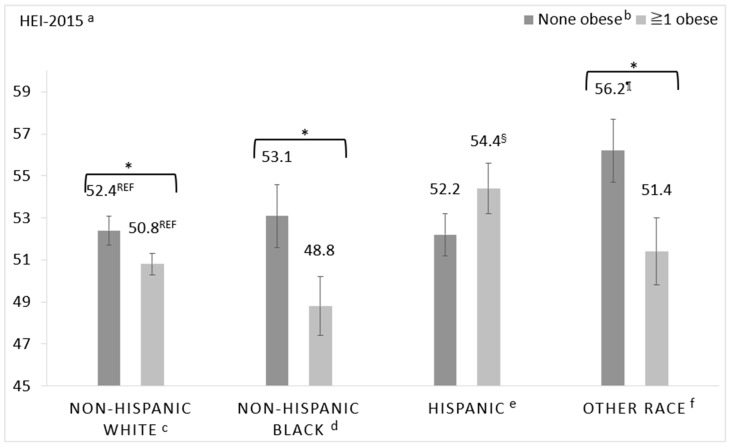
Multivariable-adjusted least square means of total HEI-2015 score of household grocery purchases of households participating in the National Household Food Acquisition and Purchase Survey 2012–2013, (*n* = 3954), stratified by race/ethnicity and weight status of household members (none obese vs. ≥1 obese). ^a^ Healthy Eating Index 2015, ^b^ obesity defined as body mass index (BMI) ≥ 30 kg/m^2^, ^c^ non-Hispanic White, none obese, *n* = 1232; non-Hispanic White, ≥1 obese, *n* = 1161, ^d^ non-Hispanic Black, none obese, *n* = 164; non-Hispanic Black, ≥1 obese, *n* = 298, ^e^ Hispanic, non-obese, *n* = 292; Hispanic, ≥1 obese, *n* = 507, ^f^ Other race, none obese, *n* = 187; Other race, ≥1 obese, *n* = 113. Scores under asterisked (*) brackets indicate significant within race/ethnicity group post hoc differences; superscripted symbols indicate significant between-groups comparison using NHW as the reference group—i.e., ¶ indicates significantly different from NHW, none obese and § indicates significantly different from NHW, ≥1 obese.

Significant interactions warranting stratification were identified between SNAP and food insecurity (*p* = 0.06), SNAP and household-level obesity (*p* = 0.04), and race/ethnicity and household-level obesity (*p* = 0.01). Interaction effects for race/ethnicity and income were non-significant. The multivariable-adjusted least square means of HEI-2015 total scores of the stratified analyses are presented in Figure 3, Figure 4 and Figure 5.

### 3.3. Associations between Food Security and SNAP-Participation on HEI-2015 Scores

In the unadjusted analysis (see Supplementary Table S1), food secure, non-SNAP-participating households (55.9 ± 0.4 points) had higher diet quality compared to food insecure, non-SNAP-participating households (51.6 ± 0.9 points, *p* < 0.001) and SNAP-participating households with both food security (50.0 ± 0.6 points, *p* < 0.001) and food insecurity (47.9 ± 0.7 points, *p* < 0.001). Among food insecure households, those participating in SNAP had higher HEI-2015 total scores compared to non-SNAP participating households (*p* = 0.016). However, after multivariable adjustment shown in Figure 3, differences in HEI-2015 total score only remained significant for food secure, non-SNAP-participating vs. food insecure, SNAP-participating households (53.9 ± 0.5 vs. 50.3 ± 0.7, *p* = 0.007). In both, the unadjusted and multivariable-adjusted model controlling for age, PIR, smoking, and education, diet quality did not differ between food secure SNAP and non-SNAP households or between food insecure SNAP and non-SNAP households. In the multivariable-adjusted linear regression (see Supplementary Table S2), both food secure and food-insecure SNAP participants had lower HEI-2015 scores than the reference group of food secure, non-SNAP participating households.

### 3.4. Associations between SNAP-Participation and Household-Level Obesity on HEI-2015 Scores

Multivariable-adjusted least square mean HEI-2015 total scores according to SNAP-participation and household-level obesity are presented in Figure 4. Final models were adjusted for the age and education of the PR and household-level smoking and PIR. Non-SNAP households without household-level obesity had significantly higher diet quality compared to all other households (i.e., non-SNAP-participating households with household-level obesity and SNAP-participating households with or without household-level obesity), in both the unadjusted and multivariable-adjusted model (unadjusted means in Supplementary Table S3). The greatest difference in HEI-2015 scores was between SNAP-participating households with household-level obesity vs. non-SNAP-participating households without household-level obesity (50.7 ± 0.7 vs. 54.6 ± 0.6 points, *p* = 0.002). While non-SNAP-participating households with household-level obesity had significantly higher HEI-2015 total scores compared to SNAP-participating households with and without household-level obesity in the unadjusted analyses (53.6 ± 0.6 vs. 48.8 ± 0.7, *p* < 0.001 and 49.5 ± 0.8, *p* = 0.003, respectively), the differences did not remain significant in the multivariable-adjusted model.

### 3.5. Associations between Race/Ethnicity and Household-Level Obesity on HEI-2015 Scores

Multivariable-adjusted least square mean HEI-2015 total scores according to race/ethnicity and household-level obesity are presented in Figure 5. Two comparisons were made: (1) within each racial/ethnic group between households with and without household-level obesity and (2) across racial/ethnic groups among households with and without household-level obesity using NHW households as the reference group. Multivariable-adjusted mean HEI-2015 total scores differed significantly between households with and without household-level obesity when the PR was NHW (50.8 ± 0.5 vs. 52.4 ± 0.7 points, *p* = 0.046), NHB (48.8 ± 1.5 vs. 53.1 ± 1.4 points, *p* = 0.018), or of Other race/ethnicity (51.4 ± 1.5 vs. 56.2 ± 1.6 points, *p* = 0.035). Differences in scores were of similar magnitude in the unadjusted model (see Supplementary Table S4). There was no difference in diet quality between Hispanic households with and without household-level obesity in either the unadjusted or multivariable adjusted models (54.4 ± 1.0 vs. 52.2 ± 1.2 points, *p* = 0.21 in the multivariable adjusted model). Among households with household-level obesity, NHW households had higher diet quality compared to NHB households in unadjusted analyses (53.0 ± 0.6, vs. 49.0 ± 1.6 points, *p* = 0.024), but not in the multivariable-adjusted model (50.8 ± 0.5 vs. 48.8 ± 1.5 points, *p* = 0.22). Hispanic households with household-level obesity had higher diet quality compared to NHW households with household-level obesity in adjusted models (54.4 ± 1.0 vs. 50.8 ± 0.5 points, *p* = 0.007).

## 4. Discussion

The present study described racial/ethnic, socioeconomic, and weight-based disparities in household FAH acquisition diet quality. Secondarily, this study descriptively evaluated whether these differences were consistent with sociodemographic disparities in diet quality observed at the individual-level. The overall mean HEI-2015 score was 54.7 out of 100, indicating that overall FAH acquisition quality needs improvement. Moreover, since these scores do not reflect FAFH, which in FoodAPS have previously been found to have 15% lower HEI-2010 scores compared to FAH [53,54,55], overall actual intake diet quality is likely lower. FAH acquisition HEI-2015 scores were also lower than individual-level HEI-2015 scores in the 2011–2012 National Health and Nutrition Examination Survey of 56.6 that reflected both FAH and FAFH items measured via 24 h recalls [29]. The lower, yet still possibly inflated, FAH HEI-15 scores may suggest that collecting dietary data primarily via a scanner is subject to less social desirability bias and/or errors related to portion estimation or recall compared to self-reported intake [56].

### 4.1. Sociodemographic Differences in FAH Acquisition Quality

Disparities in the diet quality of household food acquisitions according to SNAP participation, food security status, race/ethnicity, and the presence of obesity in the household were observed in the FoodAPS. The current analysis of the FoodAPS data suggests that many of these sociodemographic factors are intertwined and can work in confluence or divergence to influence diet quality. For example, only when SNAP participation and food insecurity co-occurred was there an association with poorer diet quality where households that reported both SNAP participation and food insecurity had lower diet quality than non-participating, food secure households. However, SNAP-participating households that reported food security did not have significantly lower diet quality than non-participating, food secure households, suggesting that when households concurrently experience food insecurity while receiving SNAP benefits, diet quality may be adversely affected.

The results observed in the present study mirror national trends in individual-level diet quality but also differ in some important ways. In a 2014 systematic review, Andreyeva et al. [49] reported that across 25 studies, diet quality was systematically lower among SNAP participants than among non-participants. However, due to differences in methodology, varied statistical analyses and controls across studies, and the age of the studies included (e.g., older studies may not reflect changes to food assistance programs in the U.S. that could impact diet quality), the authors concluded that additional research is needed using alternative sources of data (i.e., FoodAPS) to examine whether those differences remain in contemporary samples [49]. Findings from the present study complement the ongoing debate about diet quality among SNAP households [49,57], and suggest that the co-occurrence of food insecurity and SNAP participation, may exacerbate some of the observable differences between SNAP- and non-SNAP-participating households. Previous research in FoodAPS notes that the observed differences in diet quality between SNAP participating households and non-SNAP participating households likely reflect differences in age, household composition, proportion of FAH acquisitions, and education rather than SNAP participation [55]. Other research in FoodAPS found that the total HEI-2010 score for FAH acquisitions among food insecure households was approximately 10% lower than that for food secure households [58]. Taken together, additional efforts toward alleviating food insecurity among lower-income households may be needed to reduce diet-quality-related disparities [59].

Results from the current study also found that household-level obesity was associated with lower FAH acquisition quality, irrespective of SNAP participation. Both SNAP-participating households and non-SNAP-participating households with household-level obesity had lower FAH acquisition quality than non-SNAP-participating households without household-level obesity. A similar pattern was observed among racial/ethnic groups where, excluding Hispanic households, household-level obesity was associated with lower FAH acquisition quality among NHW, NHB, and Other race/ethnicity households than when obesity was absent. In contrast to some previous studies examining individual-level diet [60,61], in multivariable-adjusted analyses, no significant differences in HEI-2015 scores were observed between NHB or Hispanic households and NHW households, regardless of household-level obesity. Taken together, the finding that the overall FAH acquisition quality of households with (versus without) household-level obesity was lower among SNAP and non-SNAP households and across all racial/ethnic groups (excluding Hispanic households with obesity who had higher FAH acquisition quality than NHW households with obesity), suggests that the household-level obesity may be a risk factor for poor diet quality [62] or indicative of a less healthy food environment. Previous research in FoodAPS suggested that households with children experiencing obesity were typically located in suboptimal nutrition environments, and FAH acquisition quality may reflect these food environment barriers [63]. Moreover, the absence of differences in HEI-2015 scores between NHW and NHB adults in households with and without obesity potentially suggests that consistently observed disparities between NHW and NHB adults in individual-level studies could be driven more by FAFH. This speculation is supported by a nationally representative analysis of US young adults where total diet quality measured using the HEI-2015 was significantly higher in NHW compared to NHB adults, however, in the most recent cycle of data, diet quality of FAH was similar for NHW and NHB [64].

Among Hispanic households, however, household-level obesity was not associated with lower diet quality. Previous research has documented the “Hispanic paradox” [65], where Hispanics have lower rates of cardiovascular disease despite higher prevalence of cardiovascular risk factors including central obesity, lower socioeconomic status, diabetes, and other chronic conditions. Diet quality has been proposed as a potential explanatory factor in this paradox as traditional diets are characterized by higher intakes of cardioprotective foods such as legumes and fruits. In exploratory analyses (see Supplementary Table S4), this study found that Hispanic households with household-level obesity vs. NHW households with household-level obesity differed with respect to greens and beans, total and whole fruit, as well as sodium, refined grains, and total protein.

### 4.2. Using FAH Acquisition Data as a Proxy Measure of Dietary Intake Quality

Despite known sources of error with FAH acquisition data [66,67] and inherent differences with dietary intake data, the present study found that sociodemographic differences in diet quality measured from household-level FAH data similarly reflected findings from individual-level self-reported intake, supporting further exploration of FAH data to complement individual-level recall measures. While FAH acquisitions in FoodAPS are self-reported, data are collected prospectively and primarily using scanners, changing the nature of the error from retrospective recall methods such as 24 h dietary recalls and food frequency questionnaires. As it becomes easier to link household grocery purchase records with point-of-sale systems via customer loyalty cards, the promise of being able to collect purchase data in an automatic and objective manner and use it as a valid metric of population-level diet quality surveillance is compelling.

### 4.3. Strengths and Limitations

Conclusions drawn from the analyses of the FoodAPS data should be interpreted in the context of some limitations. This cross-sectional study collected all data between April 2012 and January 2013, so dietary data collected may not entirely reflect seasonal variation in food acquisitions that may occur in some regions in the U.S. and temporality cannot be established. Additionally, FAH acquisition data do not account for food waste, and because perishable foods tend to have higher rates of food waste than non-perishable foods, this may inflate FAH acquisition quality estimates. Lastly, the households in the sample deemed to have valid FAH acquisition data generally had higher levels of education, less food insecurity, and lower participation in SNAP, which may have attenuated differences in FAH acquisition quality between these subgroups.

There are several strengths worth mentioning as well. FoodAPS is the first nationally representative sample of household food acquisitions and purchases, and thus, the analysis presented here is generalizable to the overall dietary quality of FAH acquisitions in the U.S. Additionally, a complete food acquisition record was collected over a 7 day period, which is likely reflective of usual dietary intake. Moreover, because scanning food acquisitions relies less on memory than 24 h dietary recalls and food frequency questionnaires, self-reporting biases may be reduced or different in structure than recall methods, though this is an empirical question that requires further evaluation [66,67]. Lastly, the conceptual framework developed by the authors may provide a useful lens for evaluating individual- and environmental-level determinants of diet quality.

## 5. Conclusions

This analysis within FoodAPS suggests that dietary data from FAH acquisitions may be useful for monitoring and assessing population dietary quality. The present study drew similar conclusions as individual-level studies about disparities in household-level diet quality across racial/ethnic groups, food insecure households, and households with obesity. Automatic, prospective collection of grocery purchase data is an important next step to expand the use of FAH acquisitions to evaluate dietary quality. Access to such data would allow researchers to seamlessly evaluate individual variability and responsiveness to dietary interventions and help reduce self-report bias. The recent creation of a national Universal Purchase Code (UPC) database linked to the U.S. Department of Agriculture food codes [68] will ideally expand use of this potentially cost-effective, more objective metric of dietary quality.

## Figures and Tables

**Table 1:** Demographic, socioeconomic, and clinical characteristics of households participating in the National Household Food Acquisition and Purchase Survey 2012–2013 (*n* = 3961), according to Healthy Eating Index 2015 total score group (<40, 40–59, and ≥60) for grocery purchases for food at home.

Characteristic	All Households	HEI-2015 ^a^ <40	HEI-2015 40–59	HEI-2015 ≥60	*p*
	(*n* = 3961) Mean (SE ^b^)	(*n* = 702) Mean (SE)	(*n* = 2195) Mean (SE)	(*n* = 1064) Mean (SE)	
Percent, %		17.7	55.4	26.9	
Household size	2.49 (0.05)	2.54 (0.11)	2.59 (0.06)	2.32 (0.05)	0.005
Children (0–18 years) in HH ^c^	0.64 (0.03)	0.70 (0.07)	0.73 (0.05)	0.49 (0.03)	<0.001
Race/ethnicity of primary respondent, %					
Non-Hispanic White	70.3	69.5	69.7	71.6	<0.001
Non-Hispanic Black	9.9	11.7	12.0	5.93	
Hispanic	13.0	14.5	12.5	13.2	
Other race (non-Hispanic)	6.8	4.36	5.85	9.32	
Sex of primary respondent, %					0.59
Male	29.8	30.4	30.9	28.0	
Female	70.2	69.6	69.1	72.0	
Age primary respondent, years	50.6 (0.53)	48.4 (1.39)	50.4 (0.68)	51.8 (0.65)	0.02
Education level primary respondent, %					<0.001
Less than high school	9.1	12.0	9.93	6.54	
High school degree/some college	57.8	71.1	61.1	47.1	
Bachelor’s degree or higher	33.1	17.0	29.0	46.3	
Family income to poverty ratio, %					<0.001
<130%	16.9	27.2	18.1	10.9	
130–349%	41.1	46.8	42.6	36.4	
≥350%	42.0	25.9	39.4	52.7	
SNAP ^d^ participation, %	12.7	21.0	14.7	6.27	<0.001
WIC ^e^ participation ^f^, %	27.0	33.3	29.8	19.4	0.038
Food security status, %					<0.001
Food secure household	86.0	78.2	84.5	91.5	
Food insecure household	14.0	21.8	15.5	8.50	
Smoker in HH, %	29.3	46.8	32.9	16.4	<0.001
≥1 obese ^g^ person in HH, %	45.4	53.7	48.6	36.9	<0.001
Self-perceived health status of primary respondent, %					<0.001
Excellent	13.1	9.64	13.1	14.5	
Very good	34.5	24.2	30.8	44.6	
Good	36.0	40.7	38.7	29.7	
Fair	13.4	20.1	14.1	9.65	
Poor	3.02	5.41	3.35	1.52	
Region, %					0.001
Northeast	15.8	15.0	14.6	18.0	
Midwest	31.4	27.3	33.6	29.4	
South	34.7	44.1	35.6	29.4	
West	18.2	13.6	16.3	23.3	
HH located in rural census tract, %	34.6	40.8	37.0	28.3	0.004
Total FAH ^h^ purchases in 7 days, kcal	35615.9 (730.5)	32271.0 (1698.3)	37370.4 (1068.5)	34209.6 (1203.3)	0.99
Total FAH items purchased in 7 days	33.1 (0.58)	26.6 (1.34)	32.93 (0.79)	35.9 (0.88)	<0.001
Perceived healthfulness of diet ^i^, %					<0.001
Excellent	8.20	5.6	6.3	12.3	
Very good	29.6	23.9	26.7	36.8	
Good	42.0	39.9	45.4	37.4	
Fair	17.0	24.5	18.5	11.7	
Poor	3.11	6.03	3.15	1.83	

^a^ Healthy Eating Index 2015 for grocery purchases for food at home; ^b^ standard error; ^c^ household; ^d^ Supplemental Nutrition Assistance Program; ^e^ Special Supplemental Nutrition Program for Women, Infants, and Children (WIC); ^f^ of WIC-eligible households (*n* = 896), ^g^ body mass index (BMI) ≥ 30 kg/m^2^; ^h^ food at home; ^i^ the primary respondent’s assessment of how healthful the diet is of the overall household. All values are means ± SE unless otherwise noted. *p*-values were estimated by unadjusted linear regression, treating HEI group as an ordinal variable, for continuous variables, and by Pearson’s chi-square for categorical variables. Missing values: race of primary respondent (*n* = 4), education primary respondent (*n* = 3), SNAP participation (*n* = 1), anyone in HH receive benefits from WIC (*n* = 3100), smoking (*n* = 2), perceived healthfulness of diet (*n* = 2).

**Table 2:** Unadjusted mean Healthy Eating Index 2015 total and component scores of food-at-home purchases of households participating in the National Household Food Acquisition and Purchase Survey 2012–2013, overall and according to household characteristics (*n* = 3961).

HEI-2015 ^a^ Total and Component Scores (Maximum Score)	Mean (SE ^b^)	*p*
**All households**		
Total score (100)	54.7 (0.4)	
Total fruits (5)	2.8 (0.1)	
Whole fruits (5)	2.9 (0.0)	
Total vegetables (5)	1.9 (0.1)	
Greens and beans (5)	2.5 (0.1)	
Whole grains (10)	2.8 (0.1)	
Dairy (10)	5.3 (0.1)	
Total protein foods (5)	3.6 (0.0)	
Seafood and plant proteins (5)	2.4 (0.1)	
Fatty acids ratio (10)	5.0 (0.1)	
Refined grains (10)	6.7 (0.1)	
Sodium (10)	6.8 (0.1)	
Added sugars (10)	5.7 (0.1)	
Saturated fats (10)	6.2 (0.1)	
**Total HEI-2015 score by race/ethnicity**		**<0.001**
Non-Hispanic White, ref. ^c^	54.9 (0.5)	
Non-Hispanic Black	51.5 (1.3)	0.008
Hispanic	54.1 (0.7)	0.366
Other race (non-Hispanic)	58.1 (1.1)	0.020
**Total HEI-2015 score by food security status**		**<0.001**
Food secure household, ref.	55.4 (0.4)	
Food insecure household	50.1 (0.6)	<0.001
**Total HEI-2015 score by SNAP ^d^-participation**		**<0.001**
Household not participating in SNAP, ref.	55.5 (0.4)	
SNAP-household	49.1 (0.5)	<0.001
**Total HEI-2015 score by weight-status**		**<0.001**
None obese in household, ref.	56.2 (0.5)	
≥1 obese ^e^ person in household	52.8 (0.6)	<0.001
**Total HEI-2015 score by family income to poverty ratio**		**<0.001**
≥350%, ref.	57.1 (0.6)	
130–349%	53.8 (0.6)	<0.001
<130%	50.7 (0.7)	<0.001

^a^ Healthy Eating Index2015 for grocery purchases for food at home; ^b^ standard error; ^c^ reference group; ^d^ Supplemental Nutrition Assistance Program; ^e^ body mass index (BMI) ≥ 30 kg/m^2^. Means and *p*-values calculated using unadjusted linear regression. Missing values: race of primary respondent (*n* = 4), SNAP participation (*n* = 1).

## Supplementary Materials

The following are available online at https://www.mdpi.com/2072-6643/12/8/2354/s1, Table S1: Unadjusted model examining mean HEI scores by SNAP and food security status (*n* = 3960), Table S2: Weighted linear regression summaries examining the influence of food insecurity, SNAP participation, household-level obesity, and race/ethnicity in FoodAPS (*n* = 3960), Table S3: Unadjusted model examining mean HEI scores by SNAP and household-level obesity status (*n* = 3957), Table S4: Unadjusted model examining mean HEI scores by race/ethnicity and household-level obesity status (*n* = 3957).

## Abbreviations

BMI	Body Mass Index
FoodAPS	Food Acquisition and Purchase Survey
FAH	food at home
HEI	Healthy Eating Index
HH	household
NHB	non-Hispanic Black
NHW	non-Hispanic White
PIR	poverty income ratio
PR	primary respondent
SNAP	Supplemental Nutrition Assistance Program
WIC	Women, Infants, and Children
