# Supplementary material for: Sociodemographic Differences in the Dietary Quality of Food-at-Home Acquisitions and Purchases among Participants in the U.S. Nationally Representative Food Acquisition and Purchase Survey (FoodAPS)
Source: Nutrients. 2020 Aug 7;12(8):2354. doi: 10.3390/nu12082354 (PMC7468991; doi:10.3390/nu12082354)
Supplement: Supplementary file 1 [file nutrients-12-02354-s001.pdf]

Table S1 – Unadjusted model examining mean HEI scores by SNAP and food security status (n=3960)

HEI Category	Food Insecure		Food Secure		Model p-value
	SNAP	Non-SNAP	SNAP	Non-SNAP	
Total Score	47.9±0.7[a]	51.6±0.9[b]	50.0±0.6[a,b]	55.9±0.4[c]	<.0001
Total Vegetables	2.4±0.1[a]	2.9±0.1[a,b]	2.6±0.1[a]	3.0±0.0[b]	<.0001
Greens & Beans	1.2±0.2[a]	1.9±0.2[a,b]	1.4±0.1[a]	2.0±0.1[b]	<.0001
Total Fruit	1.7±0.1[a]	2.2±0.1[a]	2.1±0.1[a]	2.7±0.1[b]	<.0001
Whole Fruit	1.8±0.1[a]	2.1±0.2[a,b]	2.4±0.1[b]	3.0±0.1[c]	<.0001
Whole Grains	2.0±0.2[a]	2.6±0.3[a,b]	2.0±0.2[a]	3.0±0.1[b]	<.0001
Total Dairy	4.8±0.2[a]	4.9±0.3[a,b]	5.3±0.2[a,b]	5.4±0.1[b]	0.009
Total Protein	3.6±0.1	3.4±0.2	3.7±0.1	3.6±0.0	0.54
Seafood/Plant Protein	1.8±0.1[a]	2.1±0.1[a,b]	2.1±0.1[a]	2.5±0.1[b]	0.0025
Fatty Acids	4.7±0.2	5.3±0.3	4.9±0.2	5.0±0.1	0.4301
Sodium	6.5±0.2	6.3±0.3	6.2±0.2	6.8±0.1	0.043
Refined Grains	6.3±0.3	6.2±0.3	6.7±0.2	7.0±0.1	0.0103
Saturated Fats	5.3±0.3	6.1±0.3	5.3±0.2	5.7±0.1	0.0194
Added Sugar	5.8±0.2[a,b]	5.6±0.3[a,b]	5.4±0.2[a]	6.3±0.1[b]	0.0014

Estimates marked with different superscript letters are significantly different at p<0.05.

Abbreviations: Healthy Eating Index (HEI), Supplemental Nutrition Assistance Program (SNAP)

Table S2– Weighted linear regression summaries examining the influence of food insecurity, SNAP participation, household-level obesity, and race/ethnicity on Healthy Eating Index scores in FoodAPS (n=3,960)

		Unadjusted model		Adjusted model	
	Exposure levels	β (95% CI)	p-value	β (95% CI)	p-value
	**Food insecurity x SNAP**				
		Model p<0.0001 ; R^2= 3.5%		Model p<0.0001 ; R^2= 11.1%	
	Food insecure/SNAP participant	-7.77 (-9.32, -6.23)	<0.0001	-3.56 (-5.63, -1.49)	0.0014
	Food insecure/non-SNAP participant	-4.12 (-5.99, -2.25)	<0.0001	-1.43 (-3.31, 0.45)	0.1
	Food secure/SNAP participant	-5.73 (-7.21, -4.25)	<0.0001	-2.32 (-4.19, -0.46)	0.0169
	Food secure/non-SNAP participant	REF		REF	
	**SNAP x household-level obesity**				
		Model p<0.0001 ; R^2= 4.2%		Model p<0.0001 ; R^2= 11.6%	
	SNAP, HH-level obesity	-7.79 (-9.57, -6.02)	<0.0001	-3.86 (-5.85, -1.87)	0.0004
	Non-SNAP, HH-level obesity	-3.29 (-4.90, -1.67)	0.0002	-2.12 (-3.60, -0.65)	0.006
	SNAP, no HH-level obesity	-7.32 (-9.32, -5.33)	<0.0001	-3.26 (-5.40, -1.12)	0.003
	Non-SNAP, no HH-level obesity	REF		REF	
	**Race/ethnicity x household-level obesity**				
		Model p<0.0001 ; R^2= 3.5%		Model p<0.0001 ; R^2= 13.3%	
NHW	no HH-level obesity				
	HH-level obesity	-3.12 (-4.87, -1.38)	*	-1.58 (-3.12, -0.03)	*
NHB	no HH-level obesity	-1.02 (-3.86, 1.81)	¶	0.71 (-1.96, 3.39)	
	HH-level obesity	-6.79 (-10.05, -3.52)		-3.56 (-6.79, -0.41)	*
Hispanic	no HH-level obesity	-2.45 (-1.96, 3.30)	¶	-0.19 (-2.91, 2.53)	
	HH-level obesity	-1.23 (-0.40, 4.19)	§	1.99 (-0.61, 4.59)	§
Other	no HH-level obesity	4.95 (4.33, 11.82)	¶	3.84 (0.62, 7.07)	¶
	HH-level obesity	-2.33 (-2.35, 3.93)	*§	-1.02 (-4.25, 2.21)	*

Unadjusted models are adjusted for age only. Adjusted models contain age as well as the following covariates: 1) food insecurity and SNAP participation – smoking status, poverty income ratio, education, 2) SNAP participation and household-level obesity – smoking status, poverty income ratio, and education, 3) race/ethnicity and household-level obesity – SNAP participation, smoking status, poverty income ratio, education, and self-reported health status.

Abbreviations: Supplemental Nutrition Assistance Program (SNAP), household (HH), non-Hispanic White (NHW), non-Hispanic Black (NHB)

The p-value columns for race/ethnicity x household-level obesity reflect comparisons within racial/ethnic groups (e.g.) where significant differences between HH-level obesity vs non HH-level obesity is denoted with an asterisk; comparisons between groups are reflected with superscripted symbols that indicate significant between-group comparison using NHW as the reference group – i.e. ¶ indicates significantly different from NHW, no HH-level obesity and § indicates significantly different from NHW, HH-level obesity. The linear regression estimates were obtained using NHW, no HH-level obesity as the reference group; within and between group comparisons were obtained using planned contrasts.

Table S3 – Unadjusted model examining mean HEI scores by SNAP and household-level obesity status (n=3957)

HEI Category	Household-level obesity		No household-level obesity		Model p-value
	SNAP	Non-SNAP	SNAP	Non-SNAP	
Total Score	48.8±0.7[a]	53.6±0.6[b]	49.5±0.8[a]	56.9±0.6[c]	<.0001
Total Vegetables	2.5±0.1[a]	2.8±0.1[b]	2.6±0.1[a,b]	3.2±0.1[c]	<.0001
Greens & Beans	1.3±0.1[a]	1.7±0.1[b]	1.4±0.1 [a,b]	2.2±0.1 [c]	<.0001
Total Fruit	2.0±0.1[a]	2.4±0.1[b]	1.8±0.2[a]	2.7±0.1[c]	<.0001
Whole Fruit	2.2±0.1[a]	2.8±0.1[b]	2.0±0.2 [a]	3.0±0.1[b]	<.0001
Whole Grains	2.1±0.2[a]	2.8±0.1[b]	2.0±0.2 [a]	3.0±0.2[b]	<.0001
Total Dairy	5.2±0.2	5.4±0.1	4.9±0.2	5.3±0.1	0.2772
Total Protein	3.7±0.1	3.6±0.1	3.6±0.1	3.5±0.1	0.392
Seafood/Plant Protein	1.8±0.1[a]	2.3±0.1	2.1±0.2	2.5±0.1[b]	<.0001
Fatty Acids	4.7±0.2	4.9±0.2	5.0±0.3	5.1±0.1	0.3847
Sodium	6.3±0.2	6.7±0.2	6.4±0.2	6.8±0.1	0.1177
Refined Grains	6.4±0.2	6.8±0.1	6.7±0.3	6.9±0.1	0.3476
Saturated Fats	5.1±0.2[a]	5.4±0.2[a]	5.6±0.2	6.1±0.1[b]	<.0001
Added Sugar	5.6±0.2[a]	6.0±0.2	5.4±0.3[a]	6.5±0.1[b]	0.0007

Estimates marked with different superscript letters are significantly different at $p<0.05$.

Abbreviations: Healthy Eating Index (HEI), Supplemental Nutrition Assistance Program (SNAP)

Table S4 – Unadjusted model examining mean HEI scores by race/ethnicity and household-level obesity status (n=3957)

**Category**	**Non-Hispanic White**		**Non-Hispanic Black**		**Hispanic**		**Other**		**Model p-value**
	**HH obesity**	**No HH obesity**	**HH obesity**	**No HH obesity**	**HH obesity**	**No HH obesity**	**HH obesity**	**No HH obesity**	
Total Score	53.0±0.6*	56.3±0.6	49.0±1.6*§	54.9±1.4	54.6±1.0	53.5±1.3	53.5±1.5*	60.7±1.5§	0.0001
Total Vegetables	2.7±0.1*	3.1±0.1	2.4±0.2	2.9±0.2	2.9±0.1	2.9±0.1	2.9±0.3*	3.6±0.2§	<.0001
Greens & Beans	1.5±0.1*	2.1±0.1	1.2±0.2*	2.1±0.3	2.1±0.1§	2.2±0.2	2.3±0.3§	2.6±0.2	<.0001
Total Fruit	2.3±0.1*	2.7±0.1	2.1±0.2	2.2±0.3	2.8±0.1§	2.4±0.2	2.5±0.2*	3.2±0.2§	0.0003
Whole Fruit	2.7±0.1*	2.9±0.1	2.1±0.3§	2.2±0.3§	3.1±0.1§	2.9±0.2	2.8±0.3*	3.6±0.2§	0.0003
Whole Grains	2.9±0.2	3.1±0.3	2.0±0.4§	2.9±0.3	2.3±0.2§	2.3±0.3	2.3±0.4	2.2±0.4	0.0227
Total Dairy	5.6±0.2	5.7±0.1	4.4±0.3*§	3.2±0.3§	5.3±0.2*	4.4±0.4§	4.7±0.5	4.7±0.4	<.0001
Total Protein	3.5±0.1	3.5±0.1	3.9±0.1§	3.4±0.2	3.8±0.1	3.7±0.2	3.4±0.3	3.5±0.2	0.0817
Seafood/Plant Protein	2.2±0.1	2.4±0.1	2.1±0.2	2.4±0.2	2.3±0.1	2.4±0.2	2.2±0.3	3.0±0.3	0.2998
Fatty Acids	4.9±0.2	4.8±0.1	5.0±0.4*	6.7±0.4§	4.9±0.4	5.3±0.4	5.0±0.4	5.4±0.4	0.0254
Sodium	6.4±0.2	6.6±0.1	6.3±0.4*	7.3±0.3§	7.4±0.2§	6.6±0.4	7.5±0.5§	7.7±0.3§	<.0001
Refined Grains	7.0±0.1	6.9±0.2	6.6±0.3	7.0±0.4	5.9±0.3§	6.2±0.3	6.6±0.4	7.5±0.3	0.0044
Saturated Fats	5.2±0.2*	5.8±0.2	5.3±0.3*	7.0±0.3§	5.6±0.2	6.5±0.4	5.9±0.5	6.5±0.5	<.0001
Added Sugar	6.0±0.2	6.4±0.2	5.6±0.2	5.6±0.4	6.3±0.3	5.8±0.4	5.2±0.5*	7.3±0.4§	0.0003

* Designates significant differences between households with and without obesity within the racial/ethnic group

§ Designates significantly different from the non-Hispanic White referent groups

Abbreviations: household (HH), Healthy Eating Index (HEI), Supplemental Nutrition Assistance Program (SNAP)
